# 2-Oxo-4-trifluoro­meth­yl-2*H*-chromen-7-yl 2-bromo-2-methyl­propano­ate

**DOI:** 10.1107/S1600536810020234

**Published:** 2010-06-09

**Authors:** N. Haridharan, V. Ramkumar, R. Dhamodharan

**Affiliations:** a Department of Chemistry, IIT Madras, Chennai, TamilNadu, India

## Abstract

In the title compound, C_14_H_10_BrF_3_O_4_, the coumarin ring system is almost plannar (r.m.s. deviation = 0.025 Å) and a short C—H⋯F contact occurs. The propano­ate fragment is orientated almost perpendicular to the ring [dihedral angle = 71.80 (12)°]. In the crystal, mol­ecules are linked by C—H⋯O hydrogen bonds, generating [100] chains.

## Related literature

For the applications of the title compound in polymer chemistry, see: Sinkel *et al.* (2008 ▶); Matyjaszewski *et al.* (2008 ▶); Stenzel-Rosenbaum *et al.* (2001 ▶).
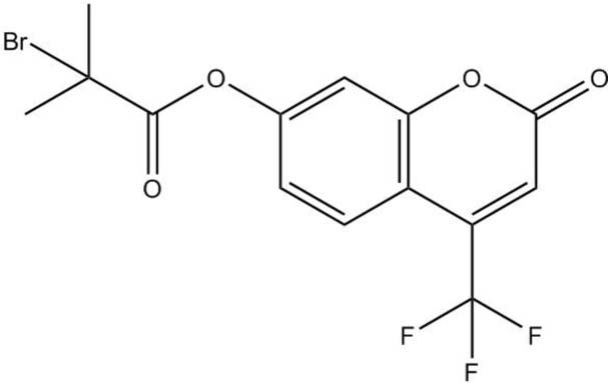


## Experimental

### 

#### Crystal data

C_14_H_10_BrF_3_O_4_
*M* *_r_* = 379.13
Triclinic, 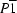

*a* = 6.1842 (4) Å
*b* = 11.0297 (6) Å
*c* = 11.0619 (7) Å
α = 99.982 (2)°
β = 91.797 (2)°
γ = 104.387 (2)°
*V* = 717.61 (8) Å^3^
*Z* = 2
Mo *K*α radiation
μ = 2.91 mm^−1^
*T* = 298 K
0.40 × 0.26 × 0.24 mm

#### Data collection

Bruker APEXII CCD diffractometer
Absorption correction: multi-scan (*SADABS*; Bruker, 2004 ▶) *T*_min_ = 0.389, *T*_max_ = 0.542
9951 measured reflections
3796 independent reflections
2390 reflections with *I* > 2σ(*I*)
*R*_int_ = 0.022

#### Refinement

*R*[*F* ^2^ > 2σ(*F* ^2^)] = 0.040
*wR*(*F* ^2^) = 0.109
*S* = 1.01
3796 reflections
201 parameters
H-atom parameters constrained
Δρ_max_ = 0.78 e Å^−3^
Δρ_min_ = −0.85 e Å^−3^

### 

Data collection: *APEX2* (Bruker, 2004 ▶); cell refinement: *SAINT-Plus* (Bruker, 2004 ▶); data reduction: *SAINT-Plus* and *XPREP* (Bruker, 2004 ▶); program(s) used to solve structure: *SHELXS97* (Sheldrick, 2008 ▶); program(s) used to refine structure: *SHELXL97* (Sheldrick, 2008 ▶); molecular graphics: *ORTEP-3* (Farrugia, 1997 ▶); software used to prepare material for publication: *SHELXL97*.

## Supplementary Material

Crystal structure: contains datablocks global, I. DOI: 10.1107/S1600536810020234/hb5450sup1.cif

Structure factors: contains datablocks I. DOI: 10.1107/S1600536810020234/hb5450Isup2.hkl

Additional supplementary materials:  crystallographic information; 3D view; checkCIF report

## Figures and Tables

**Table 1:** Hydrogen-bond geometry (Å, °)

*D*—H⋯*A*	*D*—H	H⋯*A*	*D*⋯*A*	*D*—H⋯*A*
C5—H5⋯F2	0.93	2.47	3.019 (3)	118
C6—H6⋯O4^i^	0.93	2.52	3.261 (4)	136

Symmetry code: (i) 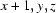.

**Fig. 1.**
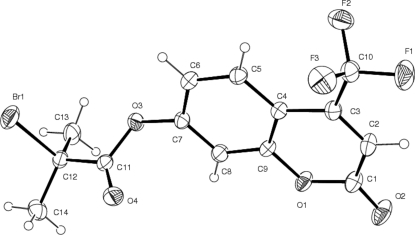
View of (I) with atoms represented as 30% probability ellipsoids.

**Fig. 2.**
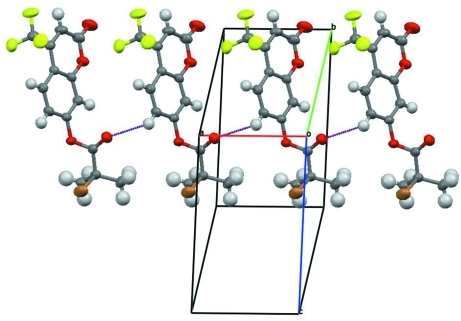
The packing diagram showing the C—H···O interaction.

## supplementary crystallographic information

### Comment

The title compound C_14_H_10_BrF_3_O_4_, is a monofunctional coumarin derivative, which is used as an initiator (Sinkel**et al.** 2008) in Atom Transfer Radical Polymerization (ATRP). Being a monofunctional unit it can form end-functionalized linear polymers (Matyjaszewski *et al.* 2008; Stenzel-Rosenbaum *et al.*2001) when used as an initiator. Since most of the synthesized functionalized initiators are characterized by other techniques, their single crystal XRD reports are few.

The title compound is one such successful ATRP initiator which was crystallised from chloroform. It contains coumarin derivative with bromo methyl propanoate. The coumarin moiety is an important oxygen containing heterocyclic compound with diverse bioactivities such as non peptidic HIV protease inhibition and tyrosine kinase inhibition. Owing to such interesting properties, the synthesis of coumarin based initiators and their crystal structures are worth while to study.

In the title compound C_14_ H_10_ Br F_3_ O_4_, the coumarin ring system is plannar with the 2-bromo-2-methyl propanoate moiety almost perpendicular. The C—F bond lengths of 1.333 (2) Å,1.324 (3)Å and 1.331 (3)Å are normal in this structure. One F atom (F1) lie in plane with the coumarin ring system and the other two F atoms are above and below the plane. The torsion angle of C6—C7—O3—C11 and C8—C7—O3—C11 are -114.21 (3)° and 71.42 (2)° respectively. The crystal is stabilized by intermolecular C—H···O hydrogen bond.

### Experimental

7-Hydroxy-4-trifluoromethylcoumarin 5 g (0.02 mole), triethylamine 4.83 g (0.04 mole) and THF (400 ml) were placed in a 3-neck round bottomed flask. Bromoisobutyrl bromide 10.9 g (0.04 mole) was added slowly, using a syringe, with stirring, upon which an white precipitate of triethylammonium bromide was formed. The mixture was left to react for 6 hours, with stirring. Subsequently, triethylammonium bromide, the precipitate was removed by filtration and the THF was removed by rotary evaporation. The resulting crude product was dissolved in ethyl acetate, washed with bicarbonate solution and then with water thrice followed by brine solution and dried over anhydrous sodium sulphate. The resulting solvent was removed by rotary evaporation. The product was purified by column chromatography technique using 15% ethyl acetate in hexane as the eluent to obtain pure initiator as a bright white solid. Recrystallization of the compound from chloroform gave colourless blocks of (I).

### Refinement

All hydrogen atoms were fixed geometrically and allowed to ride on the parent carbon atoms, with aromatic C—H = 0.93 Å and methyl C—H = 0.96 Å. The displacement parameters were set for phenyl H atoms at U_iso_(H) = 1.2U_eq_(C) and methyl H atoms at U_iso_(H) = 1.5U_eq_(C).

### Crystal data

**Table:**

C_14_H_10_BrF_3_O_4_	*Z* = 2
*M**_r_* = 379.13	*F*(000) = 376
Triclinic, *P*1	*D*_x_ = 1.755 Mg m^−^^3^
Hall symbol: -P 1	Mo *K*α radiation, λ = 0.71073 Å
*a* = 6.1842 (4) Å	Cell parameters from 3404 reflections
*b* = 11.0297 (6) Å	θ = 2.4–25.3°
*c* = 11.0619 (7) Å	µ = 2.91 mm^−^^1^
α = 99.982 (2)°	*T* = 298 K
β = 91.797 (2)°	Rectangular, colourless
γ = 104.387 (2)°	0.40 × 0.26 × 0.24 mm
*V* = 717.61 (8) Å^3^	

### Data collection

**Table:**

Bruker APEXII CCD diffractometer	3796 independent reflections
Radiation source: fine-focus sealed tube	2390 reflections with *I* > 2σ(*I*)
graphite	*R*_int_ = 0.022
phi and ω scans	θ_max_ = 30.8°, θ_min_ = 1.9°
Absorption correction: multi-scan (*SADABS*; Bruker, 2004)	*h* = −7→8
*T*_min_ = 0.389, *T*_max_ = 0.542	*k* = −15→14
9951 measured reflections	*l* = −13→15

### Refinement

**Table:**

Refinement on *F*^2^	Primary atom site location: structure-invariant direct methods
Least-squares matrix: full	Secondary atom site location: difference Fourier map
*R*[*F*^2^ > 2σ(*F*^2^)] = 0.040	Hydrogen site location: inferred from neighbouring sites
*wR*(*F*^2^) = 0.109	H-atom parameters constrained
*S* = 1.01	*w* = 1/[σ^2^(*F*_o_^2^) + (0.0464*P*)^2^ + 0.448*P*] where *P* = (*F*_o_^2^ + 2*F*_c_^2^)/3
3796 reflections	(Δ/σ)_max_ < 0.001
201 parameters	Δρ_max_ = 0.78 e Å^−^^3^
0 restraints	Δρ_min_ = −0.85 e Å^−^^3^

### Special details

**Table:**

Geometry. All esds (except the esd in the dihedral angle between two l.s. planes)are estimated using the full covariance matrix. The cell esds are takeninto account individually in the estimation of esds in distances, anglesand torsion angles; correlations between esds in cell parameters are onlyused when they are defined by crystal symmetry. An approximate (isotropic)treatment of cell esds is used for estimating esds involving l.s. planes.
Refinement. Refinement of *F*^2^ against ALL reflections. The weighted *R*-factor wR and goodness of fit *S* are based on *F*^2^, conventional *R*-factors *R* are based on *F*, with *F* set to zero for negative *F*^2^. The threshold expression of *F*^2^ > σ(*F*^2^) is used only for calculating *R*-factors(gt) etc. and is not relevant to the choice of reflections for refinement. *R*-factors based on *F*^2^ are statistically about twice as large as those based on *F*, and *R*- factors based on ALL data will be even larger.

### Fractional atomic coordinates and isotropic or equivalent isotropic displacement parameters (Å^2^)

**Table:**

	*x*	*y*	*z*	*U*_iso_*/*U*_eq_	
Br1	0.06908 (6)	−0.04175 (3)	0.29516 (4)	0.06662 (16)	
C1	0.2614 (5)	0.5339 (3)	−0.2219 (3)	0.0450 (7)	
C2	0.4147 (5)	0.4806 (3)	−0.2966 (3)	0.0450 (7)	
H2	0.4574	0.5118	−0.3675	0.054*	
C3	0.4972 (4)	0.3873 (3)	−0.2666 (2)	0.0363 (6)	
C4	0.4377 (4)	0.3379 (2)	−0.1559 (2)	0.0322 (5)	
C5	0.5183 (4)	0.2437 (3)	−0.1127 (2)	0.0375 (6)	
H5	0.6232	0.2098	−0.1557	0.045*	
C6	0.4449 (5)	0.2007 (3)	−0.0080 (3)	0.0408 (6)	
H6	0.4995	0.1385	0.0202	0.049*	
C7	0.2881 (4)	0.2518 (3)	0.0548 (2)	0.0359 (6)	
C8	0.2074 (4)	0.3459 (3)	0.0179 (2)	0.0386 (6)	
H8	0.1041	0.3801	0.0622	0.046*	
C9	0.2849 (4)	0.3880 (2)	−0.0872 (2)	0.0333 (6)	
C10	0.6453 (5)	0.3300 (3)	−0.3523 (3)	0.0464 (7)	
C11	0.0108 (4)	0.1442 (3)	0.1690 (3)	0.0361 (6)	
C12	−0.0307 (4)	0.1167 (3)	0.2974 (3)	0.0389 (6)	
C13	0.1076 (6)	0.2198 (3)	0.4010 (3)	0.0541 (8)	
H13A	0.2639	0.2317	0.3887	0.081*	
H13B	0.0784	0.1940	0.4787	0.081*	
H13C	0.0672	0.2983	0.4005	0.081*	
C14	−0.2790 (5)	0.0855 (3)	0.3145 (3)	0.0516 (8)	
H14A	−0.3331	0.1596	0.3129	0.077*	
H14B	−0.3045	0.0591	0.3923	0.077*	
H14C	−0.3569	0.0179	0.2493	0.077*	
F1	0.6893 (4)	0.3893 (2)	−0.44723 (18)	0.0775 (6)	
F2	0.8423 (3)	0.3353 (2)	−0.29621 (18)	0.0652 (5)	
F3	0.5518 (3)	0.20819 (19)	−0.39741 (17)	0.0645 (5)	
O1	0.2025 (3)	0.48421 (18)	−0.11926 (17)	0.0429 (5)	
O2	0.1777 (4)	0.6161 (2)	−0.2441 (2)	0.0648 (6)	
O3	0.2269 (3)	0.2130 (2)	0.16562 (17)	0.0452 (5)	
O4	−0.1216 (3)	0.1118 (2)	0.08271 (19)	0.0498 (5)	

### Atomic displacement parameters (Å^2^)

**Table:**

	*U*^11^	*U*^22^	*U*^33^	*U*^12^	*U*^13^	*U*^23^
Br1	0.0781 (3)	0.0645 (3)	0.0835 (3)	0.04411 (19)	0.0312 (2)	0.0426 (2)
C1	0.0603 (18)	0.0383 (16)	0.0422 (16)	0.0201 (13)	0.0037 (14)	0.0123 (13)
C2	0.0613 (18)	0.0436 (17)	0.0361 (15)	0.0170 (13)	0.0083 (13)	0.0174 (13)
C3	0.0408 (14)	0.0373 (15)	0.0311 (13)	0.0087 (11)	0.0039 (11)	0.0083 (11)
C4	0.0354 (13)	0.0320 (13)	0.0301 (13)	0.0100 (10)	0.0012 (11)	0.0066 (11)
C5	0.0398 (14)	0.0386 (15)	0.0398 (15)	0.0179 (11)	0.0082 (12)	0.0103 (12)
C6	0.0420 (15)	0.0426 (16)	0.0436 (16)	0.0157 (12)	0.0015 (13)	0.0166 (13)
C7	0.0355 (13)	0.0419 (15)	0.0314 (13)	0.0071 (11)	0.0006 (11)	0.0141 (12)
C8	0.0399 (14)	0.0448 (16)	0.0359 (14)	0.0165 (12)	0.0090 (12)	0.0114 (12)
C9	0.0371 (13)	0.0322 (13)	0.0343 (14)	0.0136 (11)	−0.0003 (11)	0.0097 (11)
C10	0.0507 (17)	0.055 (2)	0.0388 (16)	0.0182 (14)	0.0111 (13)	0.0158 (14)
C11	0.0355 (13)	0.0387 (15)	0.0409 (15)	0.0163 (11)	0.0076 (12)	0.0150 (12)
C12	0.0410 (14)	0.0400 (15)	0.0434 (16)	0.0164 (12)	0.0110 (12)	0.0184 (13)
C13	0.063 (2)	0.062 (2)	0.0375 (16)	0.0097 (16)	0.0049 (14)	0.0203 (15)
C14	0.0483 (17)	0.0514 (18)	0.060 (2)	0.0164 (14)	0.0240 (15)	0.0162 (16)
F1	0.1027 (17)	0.1013 (16)	0.0556 (12)	0.0515 (13)	0.0434 (12)	0.0444 (12)
F2	0.0437 (10)	0.0907 (15)	0.0663 (12)	0.0224 (10)	0.0112 (9)	0.0201 (11)
F3	0.0773 (13)	0.0584 (12)	0.0548 (11)	0.0235 (10)	0.0128 (10)	−0.0077 (9)
O1	0.0573 (12)	0.0422 (11)	0.0403 (11)	0.0277 (9)	0.0093 (9)	0.0146 (9)
O2	0.0941 (18)	0.0559 (14)	0.0647 (15)	0.0455 (13)	0.0132 (13)	0.0270 (12)
O3	0.0371 (10)	0.0647 (13)	0.0372 (10)	0.0071 (9)	0.0029 (8)	0.0271 (9)
O4	0.0422 (11)	0.0607 (13)	0.0453 (12)	0.0078 (9)	−0.0033 (10)	0.0161 (10)

### Geometric parameters (Å, °)

**Table:**

Br1—C12	1.990 (3)	C8—H8	0.9300
C1—O2	1.205 (3)	C9—O1	1.378 (3)
C1—O1	1.366 (3)	C10—F3	1.324 (4)
C1—C2	1.444 (4)	C10—F1	1.332 (3)
C2—C3	1.340 (4)	C10—F2	1.333 (4)
C2—H2	0.9300	C11—O4	1.181 (3)
C3—C4	1.447 (4)	C11—O3	1.368 (3)
C3—C10	1.507 (4)	C11—C12	1.520 (4)
C4—C9	1.391 (4)	C12—C14	1.513 (4)
C4—C5	1.404 (4)	C12—C13	1.529 (4)
C5—C6	1.375 (4)	C13—H13A	0.9600
C5—H5	0.9300	C13—H13B	0.9600
C6—C7	1.385 (4)	C13—H13C	0.9600
C6—H6	0.9300	C14—H14A	0.9600
C7—C8	1.373 (4)	C14—H14B	0.9600
C7—O3	1.400 (3)	C14—H14C	0.9600
C8—C9	1.382 (4)		
			
O2—C1—O1	117.5 (3)	F3—C10—F2	106.4 (3)
O2—C1—C2	125.6 (3)	F1—C10—F2	106.6 (2)
O1—C1—C2	116.9 (2)	F3—C10—C3	111.5 (2)
C3—C2—C1	121.9 (3)	F1—C10—C3	112.1 (3)
C3—C2—H2	119.1	F2—C10—C3	112.4 (2)
C1—C2—H2	119.1	O4—C11—O3	123.6 (2)
C2—C3—C4	120.6 (3)	O4—C11—C12	126.0 (2)
C2—C3—C10	119.4 (3)	O3—C11—C12	110.4 (2)
C4—C3—C10	119.9 (2)	C14—C12—C11	110.2 (2)
C9—C4—C5	117.5 (2)	C14—C12—C13	112.9 (3)
C9—C4—C3	116.5 (2)	C11—C12—C13	114.2 (2)
C5—C4—C3	126.0 (2)	C14—C12—Br1	107.64 (19)
C6—C5—C4	121.1 (2)	C11—C12—Br1	102.84 (17)
C6—C5—H5	119.4	C13—C12—Br1	108.4 (2)
C4—C5—H5	119.4	C12—C13—H13A	109.5
C5—C6—C7	118.8 (2)	C12—C13—H13B	109.5
C5—C6—H6	120.6	H13A—C13—H13B	109.5
C7—C6—H6	120.6	C12—C13—H13C	109.5
C8—C7—C6	122.4 (2)	H13A—C13—H13C	109.5
C8—C7—O3	119.5 (2)	H13B—C13—H13C	109.5
C6—C7—O3	117.8 (2)	C12—C14—H14A	109.5
C7—C8—C9	117.7 (2)	C12—C14—H14B	109.5
C7—C8—H8	121.2	H14A—C14—H14B	109.5
C9—C8—H8	121.2	C12—C14—H14C	109.5
O1—C9—C8	115.6 (2)	H14A—C14—H14C	109.5
O1—C9—C4	121.9 (2)	H14B—C14—H14C	109.5
C8—C9—C4	122.5 (2)	C1—O1—C9	122.1 (2)
F3—C10—F1	107.5 (2)	C11—O3—C7	117.3 (2)
			
O2—C1—C2—C3	−178.7 (3)	C2—C3—C10—F3	−115.9 (3)
O1—C1—C2—C3	−0.5 (4)	C4—C3—C10—F3	61.5 (3)
C1—C2—C3—C4	−1.1 (4)	C2—C3—C10—F1	4.7 (4)
C1—C2—C3—C10	176.3 (3)	C4—C3—C10—F1	−177.9 (2)
C2—C3—C4—C9	2.8 (4)	C2—C3—C10—F2	124.8 (3)
C10—C3—C4—C9	−174.5 (2)	C4—C3—C10—F2	−57.8 (3)
C2—C3—C4—C5	−177.8 (3)	O4—C11—C12—C14	−20.7 (4)
C10—C3—C4—C5	4.8 (4)	O3—C11—C12—C14	159.3 (2)
C9—C4—C5—C6	1.5 (4)	O4—C11—C12—C13	−149.0 (3)
C3—C4—C5—C6	−177.9 (3)	O3—C11—C12—C13	31.0 (3)
C4—C5—C6—C7	0.2 (4)	O4—C11—C12—Br1	93.8 (3)
C5—C6—C7—C8	−1.6 (4)	O3—C11—C12—Br1	−86.2 (2)
C5—C6—C7—O3	−175.8 (2)	O2—C1—O1—C9	178.6 (3)
C6—C7—C8—C9	1.2 (4)	C2—C1—O1—C9	0.3 (4)
O3—C7—C8—C9	175.3 (2)	C8—C9—O1—C1	−178.9 (2)
C7—C8—C9—O1	−178.8 (2)	C4—C9—O1—C1	1.6 (4)
C7—C8—C9—C4	0.6 (4)	O4—C11—O3—C7	3.3 (4)
C5—C4—C9—O1	177.5 (2)	C12—C11—O3—C7	−176.7 (2)
C3—C4—C9—O1	−3.1 (4)	C8—C7—O3—C11	71.5 (3)
C5—C4—C9—C8	−1.9 (4)	C6—C7—O3—C11	−114.2 (3)
C3—C4—C9—C8	177.5 (2)		

### Hydrogen-bond geometry (Å, °)

**Table:**

*D*—H···*A*	*D*—H	H···*A*	*D*···*A*	*D*—H···*A*
C5—H5···F2	0.93	2.47	3.019 (3)	118
C6—H6···O4^i^	0.93	2.52	3.261 (4)	136

Symmetry codes: (i) *x*+1, *y*, *z*.
